# Association between physiotherapist burnout and working environment during the coronavirus disease 2019 pandemic in Japan: A multicenter observational study

**DOI:** 10.1371/journal.pone.0275415

**Published:** 2022-09-29

**Authors:** Fumito Morisawa, Yuji Nishizaki, Yoshiki Irie, Shuko Nojiri, Takahiro Matsuo, Daiki Kobayashi, Hiroyuki Daida, Tohru Minamino, Tetsuya Takahashi

**Affiliations:** 1 Department of Cardiovascular Biology and Medicine, Juntendo University Graduate School of Medicine, Tokyo, Japan; 2 Rare Disease Medical Affairs, Pfizer Japan Inc., Tokyo, Japan; 3 Division of Medical Education, Juntendo University School of Medicine, Tokyo, Japan; 4 Medical Technology Innovation Center, Juntendo University, Tokyo, Japan; 5 Clinical Research and Trial Center, Juntendo University, Tokyo, Japan; 6 Department of Information and Computer Technology, Graduate School of Engineering, Tokyo University of Science, Tokyo, Japan; 7 Department of Infectious Diseases, St. Luke’s International Hospital, Tokyo, Japan; 8 Department of General Internal Medicine, St. Luke’s International Hospital, Tokyo, Japan; 9 Graduate School of Public Health, St. Luke’s International University, Tokyo, Japan; 10 Faculty of Health Science, Juntendo University, Tokyo, Japan; 11 Department of Physical Therapy, Faculty of Health Science, Juntendo University, Tokyo, Japan; Khulna University, BANGLADESH

## Abstract

Burnout among physiotherapists has been reported worldwide during the coronavirus disease 2019 (COVID-19) pandemic. However, no information was found on the prevalence of burnout among physiotherapists in Japan during the COVID-19 pandemic. Physiotherapists directly providing physiotherapy to patients with COVID-19 in the red zone of 487 medical facilities were evaluated for the prevalence of burnout using the Japanese version of the Maslach Burnout Inventory-General Survey (MBI-GS). The association between the presence or absence of burnout and the working environment was analyzed using logistic regression analysis. Among the 566 physiotherapists analyzed, 99 (17.5%) satisfied the MBI-GS criteria for burnout. Multivariate analysis showed that burnout was associated with the year of physiotherapy experiences [odds ratio (OR) 0.96, 95% confidence interval (CI) 0.93–0.99], feeling slight burden with infection control (OR 0.53, 95% CI 0.32–0.87), not feeling too burdened with infection control (OR 0.27, 95% CI 0.06–0.83), establishment of staffing standards for physiotherapy according to the number of beds (OR 1.80, 95% CI 1.09–2.96), and relaxation time (OR 0.49, 95% CI 0.30–0.82). Moreover, the OR increased as the self-improvement time decreased (OR 38.3, 95% CI 6.64–731). In Japan, the prevalence of burnout among physiotherapists during the COVID-19 pandemic was an intermediate value between the prevalence of burnout among physicians and nurses reported in previous studies. This study found the need to establish appropriate staffing standards for physiotherapy and support systems including secure self-improvement time and appropriate training according to physiotherapy experiences and each medical facility.

## Introduction

Throughout the coronavirus disease 2019 (COVID-19) pandemic, health care professionals (HCPs) have used all essential countermeasures to prevent and control infection [1–3]. Furthermore, HCPs have been engaging in medical care while being burdened with the thought of exposing themselves to COVID-19 at work and subsequently infecting their families [4]. The COVID-19 pandemic has increased the workload and stress of HCPs more than ever before [5], with studies regarding HCP burnout worldwide having emerged recently [6–12]. Some countries have reported a significant increase in HCP burnout compared to that before the COVID-19 pandemic [13]. Under these circumstances, the World Health Organization had suggested mental health countermeasures and psychosocial support for HCPs, team leaders, and managers at health facilities [14], emphasizing the need for establishing countermeasures to reduce the workload and stress of HCPs.

Similar to other countries, Japan had also reported HCP burnout during the COVID-19 pandemic, 50% of HCPs treating patients with COVID-19 experienced burnout, and the proportion of experiencing burnout was significantly greater than that for HCPs not treating patients with COVID-19 [15]. Moreover, one study showed that 46.8% of nurses, 36.4% of radiologists, and 36.8% of pharmacists experienced burnout, and HCPs who were not used to wearing personal protective equipment (PPE), struggling with less sleep than that before the COVID-19 pandemic, and desired to reduce their workload experienced burnout more frequently [16].

HCP burnout does not occur exclusively in a particular occupation. Notably, one study showed that burnout was also common among physiotherapists working during the COVID-19 pandemic [17]. In Portugal, 42% of physiotherapists experienced work-related or personal burnout based on the Copenhagen Burnout Inventory questionnaire during the COVID-19 pandemic, and factors affecting burnout were being female and providing physiotherapy to patients with COVID-19 [18]. However, given that only a few reports are available on the prevalence of burnout among physiotherapists during the COVID-19 pandemic, factors influencing the same have yet to be fully investigated. Furthermore, no information was found on the prevalence of burnout and its association with the working environment among physiotherapists during the COVID-19 pandemic in Japan, which suggests the need to clarify the prevalence of burnout and the influence of its factors.

Physiotherapists in other countries have provided physiotherapy to patients with COVID-19 regardless of severity [19], and physiotherapists have experienced burnout [17, 18]. Burnout was associated with work-related factors such as long-term unresolved stress at work [20]. Burnout of HCPs during the COVID-19 pandemic was associated with the working environment, such as workload and sleep time [16], appropriate PPE, and being pushed beyond training [6]. In Japan, physiotherapists have provided physiotherapy to patients with COVID-19 in the red zone [21] by referring to the guidance of physiotherapy management for COVID-19 [22]. However, physiotherapy with PPE required additional preparation, was difficult, and caused fatigue, and the time spent in the red zone increased [21]. Physiotherapists, as well as other HCPs such as nurses, have spent considerable time in contact with patients and provided physiotherapy in the working environment that they have never experienced before. We believe that Japanese physiotherapists might be experiencing burnout. We hypothesized that physiotherapists directly providing physiotherapy to patients with COVID-19 in the red zone experience burnout similar to other HCPs and the burnout is associated with factors in the working environment, such as the years of physiotherapy experience and support systems, including the establishment of staffing standards for physiotherapy according to the number of beds. The current study aimed to investigate the prevalence of burnout and its relationship with the working environment among physiotherapists directly providing physiotherapy to patients with COVID-19 in the red zone. This study aim was achieved.

## Material and methods

### Study design

This study conducted a web-based survey from March 5, 2021 to March 29, 2021 involving physiotherapists directly providing physiotherapy to patients with COVID-19 in the red zone of 487 medical facilities offering rehabilitation medicine, such as the intensivist training facility of the Japanese Society of Intensive Care Medicine, special functioning hospitals, and regional medical care support hospitals. The special functioning hospital approved by the Minister of Health, Labour and Welfare provides advanced medical care, develops advanced medical technologies, and conducts advanced medical care training [23]. The regional medical support hospital approved by prefectural governors provides medical care, including emergency medical care to patients referred by other medical institutions, such as family doctors, implements shared use of medical equipment in the region and conducts training for regional HCPs [23]. Participation in this web-based survey was voluntary. The research cooperation request form and the research explanatory document were mailed to the director of the physiotherapy department in 487 medical facilities offering rehabilitation medicine, such as the intensivist training facility of the Japanese Society of Intensive Care Medicine, special functioning hospitals, and regional medical care support hospitals among hospitals in Japan. The director of the physiotherapy department then shared these documents with all physiotherapists directly providing physiotherapy to patients with COVID-19 in the red zone and requested their cooperation by responding to the web-based survey. Physiotherapists after reading the research explanatory document and by clicking the participation consent button in the first page of the web-based survey consented to participation in the research. This study evaluated the prevalence of burnout using the Japanese version of the Maslach Burnout Inventory-General Survey (MBI-GS) and investigated the relationship between the presence or absence of burnout and the working environment.

### Survey item

Survey items related to physiotherapists’ characteristics, changes in lifestyle and personal clinical activities compared to before the COVID-19 pandemic, and working environment were investigated using the web-based survey system. The working environment covered work-related factors such as sleep time, overtime hours, vacations/holidays, physiotherapy situation for patients with COVID-19, average number of patients and patients with COVID-19, medical care fee billing for physiotherapy, fulfillment of education on infection prevention countermeasures, perceived psychological stress due to being in charge of patients with COVID-19, and desired support as a way to cope with stress. The survey items can be found in S1 Table.

### Japanese version of MBI-GS

The MBI-GS consists of 16 question items, each answered by selecting one of the 7 options (Never: 0 point; 2 or 3 times a year: 1 point; once a month: 2 points; 2 or 3 times a month: 3 points; once a week: 4 points; 2 or 3 times a week: 5 points; and every day: 6 points). The 16 questions are divided into three subscales (Exhaustion: 5 items; Cynicism: 5 items; and Professional Efficacy: 6 items), with the score of each subscale being calculated. Each subscale score is calculated by adding the scores of the question items belonging to each subscale and dividing the sum by the number of question items in the subscale [16, 24]. The Japanese version of the MBI-GS has been validated for the Japanese population [24–27]. The criteria for burnout include high Exhaustion scores and either high Cynicism scores or low Professional Efficacy scores [24, 28]. The cutoff values for burnout are as follows: An Exhaustion score of >3.5 and a Cynicism score of >3.5 or an Exhaustion score of >3.5 and a Professional Efficacy score of <2.5 [16, 29, 30].

### Statistical analysis

This study was conducted to explore factors related to burnout among physiotherapists during the COVID-19 pandemic in Japan. From the perspective of feasibility, we conducted a web survey for 487 medical facilities offering rehabilitation medicine, such as the intensivist training facility of the Japanese Society of Intensive Care Medicine, special functioning hospitals, and regional medical care support hospitals among hospitals in Japan. The dependent variable was burnout, and the independent variables were physiotherapists’ characteristics, changes in lifestyle and personal clinical activities compared with those before the COVID-19 pandemic, and working environment. Results are expressed as medians (interquartile ranges: IQR) or as numbers and proportions (%). Baseline categorical and continuous variables were compared between the groups using Chi-squared test or Fisher’s exact test and two-tailed Student’s t-test or Wilcoxon rank sum test. The relationship between burnout and the working environment was analyzed using logistic regression analysis by selecting variables that were significant during univariate analysis or clinically meaningful variables, which were selected by experienced physiotherapists and physicians involved in rehabilitation, for factors that appeared to be strongly associated with the physiotherapist’s mental health to control potential confounding effects. We deleted the outliers in all analyses. A p-value < 0.05 was considered statistically significant. All data were aggregated and analyzed using R software version 4.0.3.

### Ethics declarations

This study was approved by the Research Ethics Committee of the Faculty of Health Sciences, Juntendo University (Approval number: 20–035) and was conducted in accordance with the principles outlined in the Declaration of Helsinki and Ethical Guidelines for Medical and Health Research Involving Human Subjects. All participants reviewed the research explanatory document including data anonymization, voluntary participation, and the publication of study results prior to participation, and only participants with consent approval participated in this study.

## Results

A total of 691 physiotherapists accessed the web-based survey. After excluding 107 physiotherapists who did not complete the survey or consent to participate and 18 who had typing errors or outliers, 566 physiotherapists were ultimately analyzed. Among the 566 physiotherapists, 120 (21%) were female, 113 (20%) did not live with their families, and 188 (33%) did not have certification, such as being a certified physiotherapist or professional physiotherapist. The median IQR of each survey item was 37 (31–45) years for age, 13 (8–21) years for physiotherapy experience, 6 (6–7) h for average hours of sleep, and 3 (1–6) h for average overtime hours per week. The median IQR of survey items related to physiotherapy for patients with COVID-19 were as follows: 2 (1–3) patients for the average number of patients with COVID-19 in charge per day and 40 (30–40) min for the average physiotherapy duration per patient with COVID-19 (Table 1).

**Table 1:** Comparison of characteristics and the MBI-GS between the burnout and nonburnout groups.

		All	Burnout	Nonburnout		
		N = 566	n = 99	n = 467	p-value	
			(17.5%)	(82.5%)		
Sex					0.058	
	Male	446(79%)	71(72%)	375(80%)		
	Female	120(21%)	28(28%)	92(20%)		
Age (years)		37(31–45)	35(28–44)	37(32–45)	0.050	
Physiotherapy experience (years)		13(8–21)	12(6–20)	13(9–21)	0.029	*
Certification						
	Certified physiotherapist	180(32%)	24(24%)	156(33%)	0.075	
	Professional physiotherapist†	47(8.3%)	5(5.1%)	42(9.0%)	0.200	
	Instructor of cardiac rehabilitation	120(21%)	22(22%)	98(21%)	0.800	
	Certified respiratory therapist	282(50%)	38(38%)	244(52%)	0.012	*
	Not applicable	188(33%)	47(47%)	141(30%)	<0.001	**
Living together with their families					0.011	*
	Yes	453(80%)	70(71%)	383(82%)		
	No	113(20%)	29(29%)	84(18%)		
Average sleep time (hours)		6(6–7)	6(6–7)	6(6–7)	0.800	
Average overtime hours per week		3(1–6)	2(1–7)	3(1–6)	0.600	
Average vacations/holidays per month		8(8–9)	8(8–9)	8(8–9)	0.600	
Average number of patients in charge per day		12(9–14)	12(9–14)	12(8–14)	0.925	
Average number of patients with COVID-19 in charge per day		2(1–3)	2(1–3)	2(1–3)	0.400	
Average physiotherapy time per patients with COVID-19 (minutes)		40(30–40)	40(30–40)	40(30–40)	0.900	
MBI-GS						
	Exhaustion	18(12–25)	29(25–31)	16(11–21)	<0.001	**
	Cynicism	10(7–15)	23(13–28)	8(6–13)	<0.001	**
	Professional Efficacy	22(16–28)	17(14–23)	23(17–28)	<0.001	**

Values are presented as number (percentage) or median (interquartile range).

MBI-GS: Maslach Burnout Inventory-General Survey.

*: p < 0.05

**: p < 0.01.

†: Professional physiotherapist is a higher qualification of certified physiotherapist.

A total of 99 (17.5%) and 467 (82.5%) physiotherapists did and did not satisfy the Japanese version of the MBI-GS burnout criteria, respectively. Seventy-one (15.9%) male and 28 (23.3%) female physiotherapists experienced burnout, but no significant difference was found in the prevalence of burnout between male and female (p = 0.058). After comparing each MBI-GS subscale (Exhaustion, Cynicism, and Professional Efficacy) between the burnout and nonburnout groups, significant differences in all subscales were observed [Exhaustion: 29 (25–31) vs. 16 (11–21), p < 0.001; Cynicism: 23 (13–28) vs. 8 (6–13), p < 0.001; Professional Efficacy: 17 (14–23) vs. 23 (17–28), p < 0.001] (Table 1).

The results of the comparison between the burnout and nonburnout groups for each survey item are summarized in Tables 1 and 2, and the results of all survey items can be found in S2 Table. There was no significant difference in age between the two groups [35 (28–44) vs. 37 (32–45), p = 0.050]; however, the burnout group had significantly fewer years of physiotherapy experience than the nonburnout group [12 (6–20) vs. 13 (9–21), p = 0.029]. The burnout group had a significantly higher proportion of physiotherapists not living together with their families (29% vs. 18%, p = 0.011) and had no certification compared to the nonburnout group (47% vs. 30%, p < 0.001). Moreover, statistically significant differences were observed between the two groups in the proportion of physiotherapists who had experienced changes in eating habits (p < 0.001), sleep time (p = 0.007), and relaxation time (p = 0.006). In the question regarding personal clinical activities and stress, there was a statistically significant difference in the proportion of physiotherapists who had experienced changes in self-improvement time between the two groups (p < 0.001), and the burnout group had a significantly higher proportion of physiotherapists who desired support for reducing their overall workload as a method for coping with stress compared to the nonburnout group (62% vs. 49%, p = 0.021).

**Table 2:** Comparison of working environment between the burnout and nonburnout groups.

		All	Burnout	Nonburnout		
		N = 566	n = 99	n = 467	p-value	
			(17.5%)	(82.5%)		
Feeling of burden comparing infection control required for COVID-19 and regular physiotherapy					0.024	*
	Heavy burden	278(49%)	61(62%)	217(46%)		
	A little burden	235(42%)	34(34%)	201(43%)		
	Not quite feel burden	45(8.0%)	3(3.0%)	42(9.0%)		
	Not feel burden at all	8(1.4%)	1(1.0%)	7(1.5%)		
Medical care fee billing for physiotherapy of patients with COVID-19						
	No problem and no need to change	33(5.8%)	1(1.0%)	32(6.9%)	0.024	*
	Staffing standards for physiotherapy should be established according to the number of beds in the critical care ward.	159(28%)	38(38%)	121(26%)	0.012	*
Eating habits (comparison before the COVID-19 pandemic)					<0.001	**
	Became unhealthy	59(10%)	21(21%)	38(8.1%)		
	No change	456(81%)	72(73%)	384(82%)		
	Became healthy	51(9.0%)	6(6.1%)	45(9.6%)		
Sleep time (comparison before the COVID-19 pandemic)					0.007	**
	Decreased	53(9.4%)	17(17%)	36(7.7%)		
	No change	479(85%)	74(75%)	405(87%)		
	Increased	34(6.0%)	8(8.1%)	26(5.6%)		
Relaxation time (comparison before the COVID-19 pandemic)					0.006	**
	Decreased	194(34%)	47(47%)	147(31%)		
	No change	330(58%)	44(44%)	286(61%)		
	Increased	42(7.4%)	8(8.1%)	34(7.3%)		
Desired support as a way to cope with stress						
	Reduce overall workload	289(51%)	61(62%)	228(49%)	0.021	*
Self-improvement time (comparison before the COVID-19 pandemic)					<0.001	**
	Significantly increased	52(9.2%)	1(1.0%)	51(11%)		
	A little increased	143(25%)	20(20%)	123(26%)		
	Almost unchanged	268(47%)	51(52%)	217(46%)		
	A little decreased	70(12%)	14(14%)	56(12%)		
	Significantly decreased	33(5.8%)	13(13%)	20(4.3%)		

Values are presented as number (percentage).

*: p < 0.05

**: p < 0.01.

Multivariate analysis showed that burnout was associated with the year of physiotherapy experience [odds ratio (OR) 0.96, 95% confidence interval (CI) 0.93–0.99], physiotherapists who felt a little burdened (OR 0.53, 95% CI 0.32–0.87) or did not feel too burdened with infection control required for COVID-19 compared to regular physiotherapy (OR 0.27, 95% CI 0.06–0.83), and no change in relaxation time compared to that before COVID-19 pandemic (OR 0.49, 95% CI 0.30–0.82). However, burnout was positively associated with physiotherapists who desired to establish staffing standards for physiotherapy according to the number of beds in the critical care ward (OR 1.80, 95% CI 1.09–2.96). Furthermore, the OR increased as self-improvement time decreased after the start of the COVID-19 pandemic (OR 38.3, 95% CI 6.64–731) (Table 3).

**Table 3:** Relationship between the burnout and working environment.

		Univariate	Multivariate
		OR (95% CI)	OR (95% CI)
Sex	Female	1.61(0.97–2.61)	1.41(0.80–2.44)
Physiotherapy experience (years)		0.97(0.95–1.00)	0.96(0.93–0.99)
Living together with their families	No	1.89(1.14–3.07)	1.63(0.91–2.90)
Average sleep time (hours)		0.91(0.72–1.17)	0.85(0.64–1.12)
Feeling of burden comparing infection control required for COVID-19 and regular physiotherapy			
	Heavy burden	1 (reference)	1 (reference)
	A little burden	0.60(0.38–0.95)	0.53(0.32–0.87)
	Not quite feel burdened	0.25(0.06–0.73)	0.27(0.06–0.83)
	Not feel burdened at all	0.51(0.03–2.93)	0.57(0.03–3.97)
Staffing standards for physiotherapy should be established according to the number of beds in the critical care ward.		1.78(1.12–2.80)	1.80(1.09–2.96)
Relaxation time (comparison before the COVID-19 pandemic)			
	Decreased	1 (reference)	1 (reference)
	No change	0.48(0.30–0.76)	0.49(0.30–0.82)
	Increased	0.74(0.30–1.63)	0.70(0.26–1.73)
Desired support as a way to cope with stress			
	Reduced overall workload	1.68(1.08–2.64)	1.58(0.98–2.58)
Self-improvement time (comparison before the COVID-19 pandemic)			
	Significantly increased	1 (reference)	1 (reference)
	A little increased	8.29(1.66–151)	9.21(1.78–169)
	Almost unchanged	12.0(2.53–215)	13.1(2.68–236)
	A little decreased	12.7(2.43–235)	13.1(2.41–245)
	Significantly decreased	33.1(6.01–622)	38.3(6.64–731)

OR: Odds Ratio, CI: Confidence Interval.

## Discussion

The current study investigated the prevalence of burnout among 566 physiotherapists directly providing physiotherapy to patients with COVID-19 in the red zone. Moreover, this study compared each survey item according to the presence or absence of burnout and determined the relationship between burnout and the working environment. Notably, our findings showed that 17.5% of the physiotherapists experienced burnout and that the burnout group had a significantly higher proportion of physiotherapists who were not living with their families, had no certification and fewer years of physiotherapy experience, and desired support to reduce overall workload as a method to cope with stress compared to the nonburnout group. Moreover, statistically significant differences were observed between the burnout and nonburnout groups in the proportion of physiotherapists who had experienced changes in eating habits, sleep time, relaxation time, and self-improvement time. Multivariate analysis showed that burnout was correlated with the year of physiotherapy experience, feeling of burden due to infection control for COVID-19, the establishment of staffing physiotherapy standards, and relaxation time, with an increase in OR and a decrease in the self-improvement time.

This study, which was conducted in 2021, showed that 21% of physiotherapists were female, and 17.5% of the physiotherapists experienced burnout based on the Japanese version of the MBI-GS. In Portugal, one study, which was conducted in 2020, showed that 83% of physiotherapists were female, and 42% of the physiotherapists experienced work-related or personal burnout based on the Copenhagen Burnout Inventory questionnaire, and the factor that affected burnout was being female [18]. We believed that the difference in the prevalence of burnout between the two studies was influenced by the differences in the year of investigation, proportion of female physiotherapists, index of burnout, situation of the COVID-19 pandemic, and medical care system during the COVID-19 pandemic. Regarding the study of burnout among HCPs using the Japanese version of the MBI-GS, one study in 2020 showed that 71.5% of the participants were female and that 13.4% of the physicians and 46.8% of the nurses experienced burnout [16]. Moreover, one study, which was conducted after the first wave of the COVID-19 pandemic, showed that 77.7% of the participants were female and that 9.8% of the physicians and 29.4% of the nurses experienced burnout [30]. In Japan, the prevalence of burnout among physiotherapists during the COVID-19 pandemic was an intermediate value between the prevalence of burnout among physicians and nurses reported in previous studies using the same burnout index. The difference in the prevalence of burnout among HCPs in Japan may be influenced by the proportion of female HCPs, situation of the COVID-19 during the study period, and opportunity and spent time in contact with patients, but the prevalence of burnout among physiotherapists was by no means lower than that of other HCPs.

This study found that support according to the year of physiotherapy experience and increasing self-improvement time was necessary for physiotherapists in charge of patients with COVID-19. Throughout the COVID-19 pandemic, HCPs had experienced various stresses, such as increased workload and carrying out new work, which was usually not performed [12]. In consideration for mental and psychosocial support during the COVID-19 pandemic, the rotation of higher-stress and lower-stress work, partnership between inexperienced and more experienced HCPs, and the buddy system for support and stress monitoring have been proposed [14]. Some studies have reported a relationship between burnout and years of experience [10, 16], which suggests the need to provide the appropriate supervision and support for inexperienced physiotherapists [22]. Moreover, one study had suggested that burnout in HCPs was associated with an increase in working hours [10]. Our findings showed that the OR for burnout among physiotherapists increased with a decrease in self-improvement time after the start of the COVID-19 pandemic and the burnout was associated with the years of physiotherapy experience. Therefore, there is a need to secure self-improvement time and establish a support system that provides each physiotherapist with optimal support according to their physiotherapy experiences.

Our findings showed a positive association between burnout and the burden of new work caused by infection control required for COVID-19. Given that several physiotherapists desired support to reduce the overall workload, burnout could have been affected by the physiotherapist’s perception of increased burden caused by new work-related to infection control for COVID-19 without a decrease in the overall workload. Strict infection control during the COVID-19 pandemic, based on guidelines, has fostered a sense of psychological security among HCPs [5]. Several HCPs have actually found the guidelines useful and essential, whereas others have felt that compliance with the guidelines causes work delays and increased workload [31]. At first, a reduction in the total workload may be necessary to conduct physiotherapy similar to that before the COVID-19 pandemic without feeling excessive burden related to infection control for COVID-19. Moreover, HCPs have been required to undergo appropriate training on infection prevention, such as appropriate donning and doffing of PPE [1, 3], and training on the clinical management of patients with COVID-19 [32]. Physiotherapists have also been required to attend infection prevention training and undergo e-learning [22]. We believe that training is essential for achieving the same quality of physiotherapy as that before the COVID-19 pandemic while performing infection prevention countermeasures for COVID-19 without the associated burden. Appropriate training on infection control for COVID-19 according to the physiotherapy environment of each facility and the skills of each physiotherapist may be necessary.

The establishment of appropriate staffing standards for physiotherapy according to the number of beds in the critical care ward has been a well know approach in ensuring an optimal working environment for physiotherapists during the COVID-19 pandemic. The World Health Organization recommends assigning an appropriate number of HCPs according to the number of patients [3] and developing a staffing plan [32]. The guidance of physiotherapy management for COVID-19 also recommended an allocation plan for resources necessary for physiotherapy, such as adjusting the number of beds and physiotherapist assignment according to the number of patients with COVID-19 [22]. HCPs are required to establish a working environment necessary for COVID-19 infection control, such as the composition of working teams [33]. The same study found a relationship between the establishment of appropriate staffing standards for physiotherapy according to the number of beds in the critical care ward and burnout, suggesting the need to consider the establishment of appropriate staffing standards for physiotherapy to prevent burnout.

Currently, COVID-19 is re-expanding in some regions, indicating the need to improve and establish an appropriate working environment, as well as support systems, required by each physiotherapist. Reports have shown insufficient psychosocial support in the workplace during the COVID-19 pandemic promotes a high risk of burnout [7]. HCPs working in the COVID-19 ward have proposed to improve their current working environment, which would create room for leisure time and improve psychosocial support [33]. Our findings showed that burnout was associated with the change in relaxation time compared to that before the COVID-19 pandemic. Thus, we believe in the need for establishing the working environment and support system to secure relaxation time during the COVID-19 pandemic. On the other hand, team leaders and managers of medical facilities have demanded an improved working environment from a long-term perspective so that each HCP can flexibly adjust their work schedules and receive social support [14]. In the future, it will be necessary to formulate policies regarding appropriate staffing standards for physiotherapy according to the situation of each medical facility and establish support system for physiotherapists.

This study has several limitations worth noting. First, this study is a voluntary survey of physiotherapists directly providing physiotherapy to patients with COVID-19 in the red zone of 487 medical facilities. Therefore, our results cannot be generalized to include all medical facilities and physiotherapists who treat patients with COVID-19. Second, there is a possibility of selection bias. It is possible that physiotherapists interested in the survey contents and burnout participated, whereas those who had once left the workplace due to mental or physical reasons may not have participated. Third, given that the prevalence of burnout before the COVID-19 pandemic had not been assessed, this study cannot conclude whether the prevalence of burnout increased due to the COVID-19 pandemic. Fourth, this study did not investigate the COVID-19 infection status among physiotherapists; thus, this study cannot assess whether COVID-19 infection among physiotherapists is associated with burnout. Fifth, this study did not investigate mental health in physiotherapists using evaluation indices related to mental health such as depression; thus, this study cannot assess whether mental health affected burnout in physiotherapists. Finally, the Japanese version of MBI-GS is an index of burnout is based on self-reports, and the onset of burnout has not been evaluated by specialists.

## Conclusions

In Japan, the prevalence of burnout among physiotherapists during the COVID-19 pandemic was an intermediate value between the prevalence of burnout among physicians and nurses reported in previous studies. This study found the need to establish appropriate staffing standards for physiotherapy and support systems including secure self-improvement time and appropriate training according to physiotherapy experiences and each medical facility.

## Supporting information

S1 Table Survey items. (DOCX)

S2 Table Survey results. (DOCX)
