# Supplementary material for: Association between physiotherapist burnout and working environment during the coronavirus disease 2019 pandemic in Japan: A multicenter observational study
Source: PLoS One. 2022 Sep 29;17(9):e0275415. doi: 10.1371/journal.pone.0275415 (PMC9522274; doi:10.1371/journal.pone.0275415)
Supplement: S1 Table — (DOCX) [file pone.0275415.s001.docx]

**Association between physiotherapist burnout and working environment during the coronavirus disease 2019 pandemic in Japan: A multicenter observational study**

Fumito Morisawa, Yuji Nishizaki, Yoshiki Irie, Shuko Nojiri, Takahiro Matsuo, Daiki Kobayashi, Hiroyuki Daida, Tohru Minamino, and Tetsuya Takahashi

**S1 Table. Survey items**

| Sex |
| --- |
| Age (years) |
| Physiotherapy experience (years) |
| Certification |
| Living together with their families |
| Average sleep time (hours) |
| Average overtime hours per week |
| Average vacations/holidays per month |
| Physiotherapy situation for patients with COVID-19 |
| Physiotherapy for patients with COVID-19 (total days) |
| Average number of patients in charge per day |
| Average number of patients with COVID-19 in charge per day |
| Average physiotherapy time per patient with COVID-19 (minutes) |
| Physiotherapy prescriptions days per week for patients with COVID-19 |
| Main physiotherapy specifics for patients with COVID-19 |
| Validity of physiotherapy time (20 min) for patients with COVID-19 |
| Whether nurses can perform physiotherapy on behalf of physiotherapists in the red zone |
| Feeling of burden comparing infection control required for COVID-19 and regular physiotherapy |
| Medical care fee billing for physiotherapy of patients with COVID-19 |
| Circumstances of being in charge of patients with COVID-19 |
| Changes in compensation due to being in charge of patients with COVID-19 |
| Satisfaction with change in compensation due to being in charge of patients with COVID-19 |
| Restrictions on behavior and contact with others from the facility due to being in charge of patients with COVID-19 |
| Presence of adviser regarding the COVID-19-related work or stress |
| Fulfillment of education on infection prevention countermeasures |
| Changes in life (comparison before the COVID-19 pandemic) |
| Perceived psychological stress due to being in charge of patients with COVID-19 |
| Experience of wanting to quit or take a break from work due to stress |
| Desired support as a way to cope with stress |
| Personal clinical activities (comparison before the COVID-19 pandemic) |
| Requirements to further promote physiotherapy for patients with severe infectious diseases, such as those with COVID-19, in the future |
