# Supplementary material for: Association between physiotherapist burnout and working environment during the coronavirus disease 2019 pandemic in Japan: A multicenter observational study
Source: PLoS One. 2022 Sep 29;17(9):e0275415. doi: 10.1371/journal.pone.0275415 (PMC9522274; doi:10.1371/journal.pone.0275415)
Supplement: S2 Table — (DOCX) [file pone.0275415.s002.docx]

**Association between physiotherapist burnout and working environment during the coronavirus disease 2019 pandemic in Japan: A multicenter observational study**

Fumito Morisawa, Yuji Nishizaki, Yoshiki Irie, Shuko Nojiri, Takahiro Matsuo, Daiki Kobayashi, Hiroyuki Daida, Tohru Minamino, and Tetsuya Takahashi

**S2 Table. Survey results**

|  |  | All | Burnout | Nonburnout |  |  |
| --- | --- | --- | --- | --- | --- | --- |
|  |  | N = 566 | n = 99  (17.5%) | n = 467  (82.5%) | p-value | |
| Sex | |  |  |  | 0.058 |  |
|  | Male | 446(79%) | 71(72%) | 375(80%) |  |  |
|  | Female | 120(21%) | 28(28%) | 92(20%) |  |  |
| Age (years) | | 37(31–45) | 35(28–44) | 37(32–45) | 0.050 |  |
| Physiotherapy experience (years) | | 13(8–21) | 12(6–20) | 13(9–21) | 0.029 | * |
| Certification |  |  |  |  |  |  |
|  | Certified physiotherapist | 180(32%) | 24(24%) | 156(33%) | 0.075 |  |
|  | Professional physiotherapist† | 47(8.3%) | 5(5.1%) | 42(9.0%) | 0.200 |  |
|  | Instructor of cardiac rehabilitation | 120(21%) | 22(22%) | 98(21%) | 0.800 |  |
|  | Certified respiratory therapist | 282(50%) | 38(38%) | 244(52%) | 0.012 | * |
|  | Not applicable | 188(33%) | 47(47%) | 141(30%) | <0.001 | ** |
| Living together with their families | |  |  |  | 0.011 | * |
|  | Yes | 453(80%) | 70(71%) | 383(82%) |  |  |
|  | No | 113(20%) | 29(29%) | 84(18%) |  |  |
| Average sleep time (hours) | | 6(6–7) | 6(6–7) | 6(6–7) | 0.800 |  |
| Average overtime hours per week | | 3(1–6) | 2(1–7) | 3(1–6) | 0.600 |  |
| Average vacations/holidays per month | | 8(8–9) | 8(8–9) | 8(8–9) | 0.600 |  |
| Physiotherapy situation for patients with COVID-19 | |  |  |  | 0.700 |  |
|  | Currently | 211(37%) | 37(37%) | 174(37%) |  |  |
|  | 1 to 2 weeks ago | 93(16%) | 16(16%) | 77(16%) |  |  |
|  | 3 weeks to 1 month ago | 154(27%) | 25(25%) | 129(28%) |  |  |
|  | 2 to 3 months ago | 70(12%) | 13(13%) | 57(12%) |  |  |
|  | 4 to 6 months ago | 20(3.5%) | 6(6.1%) | 14(3.0%) |  |  |
|  | >6 months ago | 18(3.2%) | 2(2.0%) | 16(3.4%) |  |  |
| Physiotherapy for patients with COVID-19 (total days) | |  |  |  | 0.900 |  |
|  | Approximately 1 to 7 days | 130(23%) | 21(21%) | 109(23%) |  |  |
|  | Approximately 2 or 3 weeks | 125(22%) | 25(25%) | 100(21%) |  |  |
|  | Approximately 1 month | 89(16%) | 14(14%) | 75(16%) |  |  |
|  | 1 to 6 months | 139(25%) | 26(26%) | 113(24%) |  |  |
|  | >6 months | 83(15%) | 13(13%) | 70(15%) |  |  |
| Average number of patients in charge per day | | 12(9–14) | 12(9–14) | 12(8–14) | 0.925 |  |
| Average number of patients with COVID-19 in charge per day | | 2(1–3) | 2(1–3) | 2(1–3) | 0.400 |  |
| Average physiotherapy time per patient with COVID-19 (minutes) | | 40(30–40) | 40(30–40) | 40(30–40) | 0.900 |  |
| Physiotherapy prescriptions days per week for patients with COVID-19 | | 5(4–5) | 5(5–5) | 5(4–5) | 0.300 |  |
| Main physiotherapy specifics for patients with COVID-19 | |  |  |  |  |  |
|  | Posture management (including prone position) / positioning | 329(58%) | 57(58%) | 272(58%) | 0.903 |  |
|  | Early ambulation | 533(94%) | 95(96%) | 438(94%) | 0.400 |  |
|  | Basic movement practice (standing, stepping, etc.) | 515(91%) | 94(95%) | 421(90%) | 0.130 |  |
|  | Strength training | 490(87%) | 85(86%) | 405(87%) | 0.800 |  |
|  | Walking exercises | 443(78%) | 81(82%) | 362(78%) | 0.300 |  |
|  | Self-exercise instruction | 365(64%) | 66(67%) | 299(64%) | 0.600 |  |
|  | Remote training | 75(13%) | 11(11%) | 64(14%) | 0.500 |  |
|  | Respiratory physiotherapy | 230(41%) | 30(30%) | 200(43%) | 0.021 | * |
|  | Others | 44(7.8%) | 8(8.1%) | 36(7.7%) | 0.900 |  |
| Validity of physiotherapy time (20 min) for patients with COVID-19 | |  |  |  | 0.500 |  |
|  | Not enough | 396(70%) | 64(65%) | 332(71%) |  |  |
|  | Just right | 100(18%) | 21(21%) | 79(17%) |  |  |
|  | Many | 2(0.4%) | 0(0%) | 2(0.4%) |  |  |
|  | Not sure | 68(12%) | 14(14%) | 54(12%) |  |  |
| Whether nurses can perform physiotherapy on behalf of physiotherapists in the red zone | |  |  |  | 0.300 |  |
|  | Possible in all patients | 4(0.7%) | 2(2.0%) | 2(0.4%) |  |  |
|  | Possible depending on the patient | 496(88%) | 87(88%) | 409(88%) |  |  |
|  | Impossible | 60(11%) | 9(9.1%) | 51(11%) |  |  |
|  | Not sure | 6(1.1%) | 1(1.0%) | 5(1.1%) |  |  |
| Feeling of burden comparing infection control required for COVID-19 and regular physiotherapy | |  |  |  | 0.024 | * |
|  | Heavy burden | 278(49%) | 61(62%) | 217(46%) |  |  |
|  | A little burden | 235(42%) | 34(34%) | 201(43%) |  |  |
|  | Not quite feel burden | 45(8.0%) | 3(3.0%) | 42(9.0%) |  |  |
|  | Not feel burden at all | 8(1.4%) | 1(1.0%) | 7(1.5%) |  |  |
| Medical care fee billing for physiotherapy of patients with COVID-19 | |  |  |  |  |  |
|  | No problem and no need to change | 33(5.8%) | 1(1.0%) | 32(6.9%) | 0.024 | * |
|  | Raise the medical care fees unit price for physiotherapy in the red zone | 446(79%) | 83(84%) | 363(78%) | 0.200 |  |
|  | The medical care fees should be doubled | 306(54%) | 56(57%) | 250(54%) | 0.600 |  |
|  | Only physiotherapists who have taken certain training should be able to bill the medical care fee | 63(11%) | 8(8.1%) | 55(12%) | 0.300 |  |
|  | Remote physiotherapy should be covered by the medical care fees | 240(42%) | 39(39%) | 201(43%) | 0.500 |  |
|  | Staffing standards for physiotherapy should be established according to the number of beds in the critical care ward | 159(28%) | 38(38%) | 121(26%) | 0.012 | * |
| Circumstances of being in charge of patients with COVID-19 | |  |  |  | <0.001 | ** |
|  | By choice | 237(42%) | 23(23%) | 214(46%) |  |  |
|  | Order from my supervisors | 220(39%) | 56(57%) | 164(35%) |  |  |
|  | Others | 109(19%) | 20(20%) | 89(19%) |  |  |
| Changes in compensation due to being in charge of patients with COVID-19 | |  |  |  |  |  |
|  | Yes (salary increase) | 0(0%) | 0(0%) | 0(0%) | - |  |
|  | Yes (special allowance) | 417(74%) | 73(74%) | 344(74%) | 0.988 |  |
|  | Yes (Other) | 44(7.8%) | 9(9.1%) | 35(7.5%) | 0.600 |  |
|  | No | 118(21%) | 19(19%) | 99(21%) | 0.700 |  |
| Satisfaction with changes in compensation due to being in charge of patients with COVID-19 | |  |  |  | 0.500 |  |
|  | Fully satisfied | 27(6.0%) | 3(3.8%) | 24(6.5%) |  |  |
|  | Satisfied | 155(35%) | 24(30%) | 131(36%) |  |  |
|  | Neither | 171(38%) | 32(40%) | 139(38%) |  |  |
|  | Not satisfied | 77(17%) | 16(20%) | 61(17%) |  |  |
|  | Quite dissatisfied | 18(4.0%) | 5(6.2%) | 13(3.5%) |  |  |
| Restrictions on behavior and contact with others from the facility due to being in charge of patients with COVID-19 | |  |  |  | 0.800 |  |
|  | Yes (restriction on behavior and contact) | 130(23%) | 24(24%) | 106(23%) |  |  |
|  | Yes (only restriction on contact) | 42(7.4%) | 8(8.1%) | 34(7.3%) |  |  |
|  | Yes (only restriction on behavior) | 111(20%) | 22(22%) | 89(19%) |  |  |
|  | None | 283(50%) | 45(45%) | 238(51%) |  |  |
| Presence of adviser regarding the COVID-19-related work or stress | |  |  |  | 0.300 |  |
|  | Yes | 509(90%) | 86(87%) | 423(91%) |  |  |
|  | No | 57(10%) | 13(13%) | 44(9.4%) |  |  |
| Adviser regarding COVID-19-related work or stress | |  |  |  |  |  |
|  | Colleague | 390(77%) | 69(80%) | 321(76%) | 0.400 |  |
|  | Supervisor | 333(65%) | 52(60%) | 281(66%) | 0.300 |  |
|  | Health care department | 34(6.7%) | 3(3.5%) | 31(7.3%) | 0.200 |  |
|  | Physiotherapist at another hospital | 74(15%) | 12(14%) | 62(15%) | 0.900 |  |
|  | Family | 269(53%) | 49(57%) | 220(52%) | 0.400 |  |
|  | Friend | 83(16%) | 18(21%) | 65(15%) | 0.200 |  |
|  | Others | 21(4.1%) | 4(4.7%) | 17(4.0%) | 0.800 |  |
| Fulfillment of education on infection prevention countermeasures | |  |  |  | 0.023 | * |
|  | Underwent regular education before and new education on COVID-19 this time | 443(78%) | 69(70%) | 374(80%) |  |  |
|  | Underwent regular education before but not new education on COVID-19 this time | 43(7.6%) | 8(8.1%) | 35(7.5%) |  |  |
|  | Hardly underwent any education on infection prevention so far but took new education on COVID-19 this time | 70(12%) | 17(17%) | 53(11%) |  |  |
|  | Hardly took any education on infection prevention so far, and did not take particular education in this time | 5(0.9%) | 3(3.0%) | 2(0.4%) |  |  |
|  | Others | 5(0.9%) | 2(2.0%) | 3(0.6%) |  |  |
| Understanding of surrounding people, such as family, regarding being in charge of patients with COVID-19 | |  |  |  | 0.400 |  |
|  | Gained understanding | 392(69%) | 63(64%) | 329(70%) |  |  |
|  | Difficulty in understanding others but finally did so | 94(17%) | 18(18%) | 76(16%) |  |  |
|  | Never gained understanding of others and worked secretly | 15(2.7%) | 4(4.0%) | 11(2.4%) |  |  |
|  | Did not communicate because I thought did not gain the understanding of others | 30(5.3%) | 5(5.1%) | 25(5.4%) |  |  |
|  | Do not have any family or dependents that needed to be understood. | 35(6.2%) | 9(9.1%) | 26(5.6%) |  |  |
| Changes in lifestyle (comparison before the COVID-19 pandemic) | |  |  |  |  |  |
| Workload | |  |  |  | 0.200 |  |
|  | Decreased | 187(33%) | 40(40%) | 147(31%) |  |  |
|  | No change | 284(50%) | 44(44%) | 240(51%) |  |  |
|  | Increased | 95(17%) | 15(15%) | 80(17%) |  |  |
| Eating habits | |  |  |  | <0.001 | ** |
|  | Became unhealthy | 59(10%) | 21(21%) | 38(8.1%) |  |  |
|  | No change | 456(81%) | 72(73%) | 384(82%) |  |  |
|  | Became healthy | 51(9.0%) | 6(6.1%) | 45(9.6%) |  |  |
| Sleep time | |  |  |  | 0.007 | ** |
|  | Decreased | 53(9.4%) | 17(17%) | 36(7.7%) |  |  |
|  | No change | 479(85%) | 74(75%) | 405(87%) |  |  |
|  | Increased | 34(6.0%) | 8(8.1%) | 26(5.6%) |  |  |
| Amount of alcohol | |  |  |  | 0.800 |  |
|  | Decreased | 89(16%) | 17(17%) | 72(15%) |  |  |
|  | No change | 365(64%) | 61(62%) | 304(65%) |  |  |
|  | Increased | 112(20%) | 21(21%) | 91(19%) |  |  |
| Relaxation time | |  |  |  | 0.006 | ** |
|  | Decreased | 194(34%) | 47(47%) | 147(31%) |  |  |
|  | No change | 330(58%) | 44(44%) | 286(61%) |  |  |
|  | Increased | 42(7.4%) | 8(8.1%) | 34(7.3%) |  |  |
| Perceived psychological stress due to being in charge of patients with COVID-19 | |  |  |  |  |  |
|  | Infection to family and surroundings | 501(89%) | 87(88%) | 414(89%) | 0.800 |  |
|  | Infection to patients | 363(64%) | 68(69%) | 295(63%) | 0.300 |  |
|  | Infection to myself | 465(82%) | 83(84%) | 382(82%) | 0.600 |  |
|  | Infection to friends and colleagues | 369(65%) | 73(74%) | 296(63%) | 0.049 | * |
|  | Being in charge against my will | 27(4.8%) | 8(8.1%) | 19(4.1%) | 0.110 |  |
|  | Unfamiliar with personal protective equipment | 157(28%) | 29(29%) | 128(27%) | 0.700 |  |
|  | Prejudice from the surroundings | 157(28%) | 33(33%) | 124(27%) | 0.200 |  |
|  | Others | 35(6.2%) | 8(8.1%) | 27(5.8%) | 0.400 |  |
| Experience of wanting to quit or take a break from work due to stress | |  |  |  | <0.001 | ** |
|  | Yes | 83(15%) | 31(31%) | 52(11%) |  |  |
|  | Almost unchanged | 465(82%) | 59(60%) | 406(87%) |  |  |
|  | Not sure | 18(3.2%) | 9(9.1%) | 9(1.9%) |  |  |
| Desired support as a way to cope with stress | |  |  |  |  |  |
|  | Reduce overall workload | 289(51%) | 61(62%) | 228(49%) | 0.021 | * |
|  | Increase staff | 206(36%) | 38(38%) | 168(36%) | 0.700 |  |
|  | Gratitude and respect from others | 143(25%) | 28(28%) | 115(25%) | 0.400 |  |
|  | Danger pay | 405(72%) | 70(71%) | 335(72%) | 0.800 |  |
|  | Proper education to prevent infection, increased educational opportunities, access to educational resources | 197(35%) | 27(27%) | 170(36%) | 0.083 |  |
|  | Child care support | 40(7.1%) | 7(7.1%) | 33(7.1%) | 0.999 |  |
|  | Counseling | 57(10%) | 13(13%) | 44(9.4%) | 0.300 |  |
|  | Others | 45(8.0%) | 8(8.1%) | 37(7.9%) | 0.958 |  |
| Personal clinical activities (comparison before the COVID-19 pandemic) | |  |  |  |  |  |
| Medical care fee billing unit | |  |  |  | 0.913 |  |
|  | Significantly increased | 9(1.6%) | 2(2.0%) | 7(1.5%) |  |  |
|  | A little increased | 54(9.5%) | 8(8.1%) | 46(9.9%) |  |  |
|  | Almost unchanged | 195(34%) | 34(34%) | 161(34%) |  |  |
|  | A little decreased | 206(36%) | 35(35%) | 171(37%) |  |  |
|  | Significantly decreased | 102(18%) | 20(20%) | 82(18%) |  |  |
| Patients in charge | |  |  |  | 0.800 |  |
|  | Significantly increased | 13(2.3%) | 2(2.0%) | 11(2.4%) |  |  |
|  | A little increased | 58(10%) | 13(13%) | 45(9.6%) |  |  |
|  | Almost unchanged | 213(38%) | 38(38%) | 175(37%) |  |  |
|  | A little decreased | 217(38%) | 35(35%) | 182(39%) |  |  |
|  | Significantly decreased | 65(11%) | 11(11%) | 54(12%) |  |  |
| My own hand sanitizer | |  |  |  | 0.943 |  |
|  | Significantly increased | 430(76%) | 75(76%) | 355(76%) |  |  |
|  | A little increased | 110(19%) | 19(19%) | 91(19%) |  |  |
|  | Almost unchanged | 25(4.4%) | 5(5.1%) | 20(4.3%) |  |  |
|  | A little decreased | 1(0.2%) | 0(0%) | 1(0.2%) |  |  |
|  | Significantly decreased | 0(0%) | 0(0%) | 0(0%) |  |  |
| Disinfection of rehabilitation rooms and equipment | |  |  |  | 0.600 |  |
|  | Significantly increased | 405(72%) | 66(67%) | 339(73%) |  |  |
|  | A little increased | 124(22%) | 27(27%) | 97(21%) |  |  |
|  | Almost unchanged | 35(6.2%) | 6(6.1%) | 29(6.2%) |  |  |
|  | A little decreased | 1(0.2%) | 0(0%) | 1(0.2%) |  |  |
|  | Significantly decreased | 1(0.2%) | 0(0%) | 1(0.2%) |  |  |
| Conference in the rehabilitation department | |  |  |  | 0.900 |  |
|  | Significantly increased | 15(2.7%) | 3(3.0%) | 12(2.6%) |  |  |
|  | A little increased | 58(10%) | 8(8.1%) | 50(11%) |  |  |
|  | Almost unchanged | 256(45%) | 43(43%) | 213(46%) |  |  |
|  | A little decreased | 137(24%) | 27(27%) | 110(24%) |  |  |
|  | Significantly decreased | 100(18%) | 18(18%) | 82(18%) |  |  |
| Conference with other departments | |  |  |  | 0.500 |  |
|  | Significantly increased | 14(2.5%) | 1(1.0%) | 13(2.8%) |  |  |
|  | A little increased | 62(11%) | 11(11%) | 51(11%) |  |  |
|  | Almost unchanged | 240(42%) | 36(36%) | 204(44%) |  |  |
|  | A little decreased | 172(30%) | 35(35%) | 137(29%) |  |  |
|  | Significantly decreased | 78(14%) | 16(16%) | 62(13%) |  |  |
| Move time in the hospital | |  |  |  | 0.032 | * |
|  | Significantly increased | 32(5.7%) | 7(7.1%) | 25(5.4%) |  |  |
|  | A little increased | 81(14%) | 23(23%) | 58(12%) |  |  |
|  | Almost unchanged | 361(64%) | 51(52%) | 310(66%) |  |  |
|  | A little decreased | 61(11%) | 13(13%) | 48(10%) |  |  |
|  | Significantly decreased | 31(5.5%) | 5(5.1%) | 26(5.6%) |  |  |
| Overtime work | |  |  |  | 0.200 |  |
|  | Significantly increased | 23(4.1%) | 8(8.1%) | 15(3.2%) |  |  |
|  | A little increased | 115(20%) | 23(23%) | 92(20%) |  |  |
|  | Almost unchanged | 279(49%) | 44(44%) | 235(50%) |  |  |
|  | A little decreased | 114(20%) | 20(20%) | 94(20%) |  |  |
|  | Significantly decreased | 35(6.2%) | 4(4.0%) | 31(6.6%) |  |  |
| Internal online meetings | |  |  |  | 0.300 |  |
|  | Significantly increased | 125(22%) | 15(15%) | 110(24%) |  |  |
|  | A little increased | 177(31%) | 33(33%) | 144(31%) |  |  |
|  | Almost unchanged | 257(45%) | 50(51%) | 207(44%) |  |  |
|  | A little decreased | 0(0%) | 0(0%) | 0(0%) |  |  |
|  | Significantly decreased | 7(1.2%) | 1(1.0%) | 6(1.3%) |  |  |
| External online meeting | |  |  |  | 0.006 | ** |
|  | Significantly increased | 217(38%) | 27(27%) | 190(41%) |  |  |
|  | A little increased | 203(36%) | 34(34%) | 169(36%) |  |  |
|  | Almost unchanged | 139(25%) | 35(35%) | 104(22%) |  |  |
|  | A little decreased | 2(0.4%) | 1(1.0%) | 1(0.2%) |  |  |
|  | Significantly decreased | 5(0.9%) | 2(2.0%) | 3(0.6%) |  |  |
| Confirmation of patient’s behavior and physical condition | |  |  |  | 0.500 |  |
|  | Significantly increased | 183(32%) | 27(27%) | 156(33%) |  |  |
|  | A little increased | 209(37%) | 40(40%) | 169(36%) |  |  |
|  | Almost unchanged | 174(31%) | 32(32%) | 142(30%) |  |  |
|  | A little decreased | 0(0%) | 0(0%) | 0(0%) |  |  |
|  | Significantly decreased | 0(0%) | 0(0%) | 0(0%) |  |  |
| Remote patient support | |  |  |  | 0.200 |  |
|  | Significantly increased | 21(3.7%) | 3(3.0%) | 18(3.9%) |  |  |
|  | A little increased | 77(14%) | 10(10%) | 67(14%) |  |  |
|  | Almost unchanged | 459(81%) | 83(84%) | 376(81%) |  |  |
|  | A little decreased | 1(0.2%) | 1(1.0%) | 0(0%) |  |  |
|  | Significantly decreased | 8(1.4%) | 2(2.0%) | 6(1.3%) |  |  |
| My awareness of infection prevention | |  |  |  | 0.700 |  |
|  | Significantly increased | 446(79%) | 75(76%) | 371(79%) |  |  |
|  | A little increased | 110(19%) | 22(22%) | 88(19%) |  |  |
|  | Almost unchanged | 10(1.8%) | 2(2.0%) | 8(1.7%) |  |  |
|  | A little decreased | 0(0%) | 0(0%) | 0(0%) |  |  |
|  | Significantly decreased | 0(0%) | 0(0%) | 0(0%) |  |  |
| Self-improvement time | |  |  |  | <0.001 | ** |
|  | Significantly increased | 52(9.2%) | 1(1.0%) | 51(11%) |  |  |
|  | A little increased | 143(25%) | 20(20%) | 123(26%) |  |  |
|  | Almost unchanged | 268(47%) | 51(52%) | 217(46%) |  |  |
|  | A little decreased | 70(12%) | 14(14%) | 56(12%) |  |  |
|  | Significantly decreased | 33(5.8%) | 13(13%) | 20(4.3%) |  |  |
| Staff who have lost their positivity | |  |  |  | 0.081 |  |
|  | Significantly increased | 16(2.8%) | 5(5.1%) | 11(2.4%) |  |  |
|  | A little increased | 110(19%) | 24(24%) | 86(18%) |  |  |
|  | Almost unchanged | 426(75%) | 67(68%) | 359(77%) |  |  |
|  | A little decreased | 10(1.8%) | 1(1.0%) | 9(1.9%) |  |  |
|  | Significantly decreased | 4(0.7%) | 2(2.0%) | 2(0.4%) |  |  |
| Staff who got sick | |  |  |  | 0.700 |  |
|  | Significantly increased | 14(2.5%) | 3(3.0%) | 11(2.4%) |  |  |
|  | A little increased | 153(27%) | 30(30%) | 123(26%) |  |  |
|  | Almost unchanged | 348(61%) | 56(57%) | 292(63%) |  |  |
|  | A little decreased | 40(7.1%) | 7(7.1%) | 33(7.1%) |  |  |
|  | Significantly decreased | 11(1.9%) | 3(3.0%) | 8(1.7%) |  |  |
| Severity of the patient’s disability | |  |  |  | 0.300 |  |
|  | Significantly increased | 22(3.9%) | 1(1.0%) | 21(4.5%) |  |  |
|  | A little increased | 94(17%) | 13(13%) | 81(17%) |  |  |
|  | Almost unchanged | 446(79%) | 85(86%) | 361(77%) |  |  |
|  | A little decreased | 2(0.4%) | 0(0%) | 2(0.4%) |  |  |
|  | Significantly decreased | 2(0.4%) | 0(0%) | 2(0.4%) |  |  |
| Increasing number of patients who are less motivated or less active in rehabilitation | |  |  |  | 0.300 |  |
|  | Significantly increased | 4(0.7%) | 1(1.0%) | 3(0.6%) |  |  |
|  | A little increased | 47(8.3%) | 9(9.1%) | 38(8.1%) |  |  |
|  | Almost unchanged | 239(42%) | 50(51%) | 189(40%) |  |  |
|  | A little decreased | 165(29%) | 24(24%) | 141(30%) |  |  |
|  | Significantly decreased | 111(20%) | 15(15%) | 96(21%) |  |  |
| Increasing number of patients limiting outpatient visits due to infection risk | |  |  |  | 0.040 | * |
|  | Significantly increased | 119(21%) | 12(12%) | 107(23%) |  |  |
|  | A little increased | 262(46%) | 45(45%) | 217(46%) |  |  |
|  | Almost unchanged | 121(21%) | 29(29%) | 92(20%) |  |  |
|  | A little decreased | 42(7.4%) | 7(7.1%) | 35(7.5%) |  |  |
|  | Significantly decreased | 22(3.9%) | 6(6.1%) | 16(3.4%) |  |  |
| Increasing the number of patients wishing to be discharged due to restrictions on family visits | |  |  |  | 0.140 |  |
|  | Significantly increased | 58(10%) | 7(7.1%) | 51(11%) |  |  |
|  | A little increased | 216(38%) | 44(44%) | 172(37%) |  |  |
|  | Almost unchanged | 164(29%) | 32(32%) | 132(28%) |  |  |
|  | A little decreased | 86(15%) | 8(8.1%) | 78(17%) |  |  |
|  | Significantly decreased | 42(7.4%) | 8(8.1%) | 34(7.3%) |  |  |
| Increasing number of patients who are nervous about nosocomial infections and wish to be discharged early | |  |  |  | 0.958 |  |
|  | Significantly increased | 13(2.3%) | 2(2.0%) | 11(2.4%) |  |  |
|  | A little increased | 144(25%) | 28(28%) | 116(25%) |  |  |
|  | Almost unchanged | 211(37%) | 37(37%) | 174(37%) |  |  |
|  | A little decreased | 146(26%) | 23(23%) | 123(26%) |  |  |
|  | Significantly decreased | 52(9.2%) | 9(9.1%) | 43(9.2%) |  |  |
| Increasing number of patients who do not want to be discharged because “hospitals are rather safe” | |  |  |  | 0.700 |  |
|  | Significantly increased | 7(1.2%) | 2(2.0%) | 5(1.1%) |  |  |
|  | A little increased | 67(12%) | 13(13%) | 54(12%) |  |  |
|  | Almost unchanged | 224(40%) | 40(40%) | 184(39%) |  |  |
|  | A little decreased | 191(34%) | 34(34%) | 157(34%) |  |  |
|  | Significantly decreased | 77(14%) | 10(10%) | 67(14%) |  |  |
| Increasing families more nervous about infectious diseases than patients | |  |  |  | 0.914 |  |
|  | Significantly increased | 64(11%) | 10(10%) | 54(12%) |  |  |
|  | A little increased | 215(38%) | 40(40%) | 175(37%) |  |  |
|  | Almost unchanged | 177(31%) | 28(28%) | 149(32%) |  |  |
|  | A little decreased | 90(16%) | 17(17%) | 73(16%) |  |  |
|  | Significantly decreased | 20(3.5%) | 4(4.0%) | 16(3.4%) |  |  |
| Increasing number of patients with severe disabilities after the spread of COVID-19 | |  |  |  | 0.400 |  |
|  | Significantly increased | 23(4.1%) | 1(1.0%) | 22(4.7%) |  |  |
|  | A little increased | 87(15%) | 14(14%) | 73(16%) |  |  |
|  | Almost unchanged | 259(46%) | 52(53%) | 207(44%) |  |  |
|  | A little decreased | 147(26%) | 23(23%) | 124(27%) |  |  |
|  | Significantly decreased | 50(8.8%) | 9(9.1%) | 41(8.8%) |  |  |
| Increasing number of patients with mild disabilities after the spread of COVID-19 | |  |  |  | 0.500 |  |
|  | Significantly increased | 4(0.7%) | 1(1.0%) | 3(0.6%) |  |  |
|  | A little increased | 30(5.3%) | 8(8.1%) | 22(4.7%) |  |  |
|  | Almost unchanged | 294(52%) | 53(54%) | 241(52%) |  |  |
|  | A little decreased | 166(29%) | 27(27%) | 139(30%) |  |  |
|  | Significantly decreased | 72(13%) | 10(10%) | 62(13%) |  |  |
| Decrease in the rate of return home after the spread of COVID-19 | |  |  |  | 0.400 |  |
|  | Significantly increased | 10(1.8%) | 4(4.0%) | 6(1.3%) |  |  |
|  | A little increased | 65(11%) | 12(12%) | 53(11%) |  |  |
|  | Almost unchanged | 281(50%) | 46(46%) | 235(50%) |  |  |
|  | A little decreased | 152(27%) | 28(28%) | 124(27%) |  |  |
|  | Significantly decreased | 58(10%) | 9(9.1%) | 49(10%) |  |  |
| Increasing the number of patients who have difficulty in adjusting transfer due to the intention of the transfer hospital after the spread of COVID-19 | |  |  |  | 0.300 |  |
|  | Significantly increased | 209(37%) | 28(28%) | 181(39%) |  |  |
|  | A little increased | 240(42%) | 46(46%) | 194(42%) |  |  |
|  | Almost unchanged | 86(15%) | 19(19%) | 67(14%) |  |  |
|  | A little decreased | 23(4.1%) | 4(4.0%) | 19(4.1%) |  |  |
|  | Significantly decreased | 8(1.4%) | 2(2.0%) | 6(1.3%) |  |  |
| Requirements to further promote physiotherapy for patients with severe infectious diseases, such as those with COVID-19, in the future | |  |  |  |  |  |
|  | Workforce | 402(71%) | 76(77%) | 326(70%) | 0.200 |  |
|  | Understanding of physiatrist | 140(25%) | 27(27%) | 113(24%) | 0.500 |  |
|  | Understanding of the rehabilitation profession manager | 204(36%) | 32(32%) | 172(37%) | 0.400 |  |
|  | Hospital owners’ trust in the rehabilitation department | 277(49%) | 47(47%) | 230(49%) | 0.700 |  |
|  | Awareness-raising activities by the Japanese Physical Therapy Association | 173(31%) | 31(31%) | 142(30%) | 0.900 |  |
|  | Establishment of evidence for physiotherapy | 409(72%) | 68(69%) | 341(73%) | 0.400 |  |
|  | Increase opportunities to learn about physiotherapy for critically ill patients including infectious disease | 364(64%) | 59(60%) | 305(65%) | 0.300 |  |
|  | Physiotherapy qualification system for critically ill patients | 130(23%) | 21(21%) | 109(23%) | 0.600 |  |
|  | Others | 42(7.4%) | 6(6.1%) | 36(7.7%) | 0.600 |  |
| Values are presented as number (percentage) or median (interquartile range). | | | | | | |
| * : p < 0.05, ** : p < 0.01 | | | | | | |
| †: Professional physiotherapist is a higher qualification of certified physiotherapist. | | | | | | |
